# The prevalence and impact of self-reported foot and ankle pain in the over 55 age group: a secondary data analysis from a large community sample

**DOI:** 10.1186/s13047-019-0363-9

**Published:** 2019-11-15

**Authors:** Anne-Maree Keenan, Chris Drake, Philip G. Conaghan, Alan Tennant

**Affiliations:** 1 NIHR Leeds Biomedical Research Centre, Leeds Teaching Hospitals Trust, School of Healthcare, University of Leeds, Leeds, England; 2 Mid Yorkshire Hospitals NHS Trust, NIHR Leeds Biomedical Research Centre, Leeds, England; 3 Leeds Institute of Rheumatic and Musculoskeletal Medicine, NIHR Leeds Biomedical Research Centre, Leeds Teaching Hospitals Trust, University of Leeds, Leeds, England; 4 Leeds Institute of Rheumatic and Musculoskeletal Medicine, University of Leeds, Leeds, England

**Keywords:** Foot pain, Joint pain, Musculoskeletal pain, Functional ability

## Abstract

**Background:**

While the prevalence and impact of musculoskeletal problems are high, most attention has been directed towards the back, knee and hip disorders. Foot pain is known to be common in older adults and accounts for a significant burden on health services. The aim of this study was to assess the impact of foot and ankle joint pain, considering age, presence of co-morbidities and other site joint pain, in a large community sample.

**Methods:**

In the North Yorkshire Health study, 16,222 people over 55 years participated in a detailed survey of the prevalence and impact of lower limb joint problems. Self-assessment of overall body pain and functional activities of daily living were assessed. Participants indicated the presence of joint pain, stiffness or swelling during the last 3 months which had lasted for more than 6 weeks on a manikin: data were captured on the foot and the ankle.

**Results:**

The prevalence of self-reported foot and ankle joint pain was substantial: 184.33 per 1000, second only to knee problems. While foot pain was common, it was mostly associated with joint pain at other sites; only 1 in 11 of those with foot and ankle pain reported it only in the foot. Logistic regression modeling revealed while established factors such as co-morbidities, knee and hip problems contributed to functional impairment, foot and ankle problems contributed to an additional increased risk of having difficulty standing and walking by two fold (OR = 2.314, 95%CI 2.061–2.598), going up and down stairs by 71% (OR = 1.711, 95%CI 1.478–1.980) and getting up from a seated position by 44% (OR = 1.438, 95%CI 1.197–1.729).

**Conclusion:**

These results suggest that not only are foot problems in the over 55 age group extremely prevalent, they have a considerable impact on functional abilities.

## Background

Musculoskeletal disease presents a significant global burden and is the second leading cause of years lost to disability [1] . While the prevalence, impact and future burden of back, knee and hip pain have been well reported, equivalent data on foot pain have been limited until relatively recently. Foot pain is now known to be highly prevalent in older adults in the general community, and more common in females [2–5]. Body mass index (BMI) appears to be a strong predictor of foot pain [6, 7], with other predictors including, arthritis and previous foot pain [6]. The burden on health services is high, with foot and ankle complaints accounting for between 3 and 8% of UK primary care musculoskeletal consultations [8, 9]. However, the impact and burden of foot and ankle joint pain on activities of daily living has yet to be determined.

In the 1990s, a regional UK study was performed to determine the prevalence of knee problems in people over age 55 in the community; results of this survey on the prevalence and impact of hip and knee pain and multiple site joint pain have been previously reported [10–12]. The aim of this current study was therefore, undertaking further secondary analysis of the data, to determine the prevalence of self-reported foot and ankle joint pain in the community and its impact on important functional capabilities when present alone, or in conjunction with other common factors which also affect the activities of daily living, include co-morbidities and joint pain in other anatomical locations. Previous publications from this data have included the prevalence and impact of hip [10], knee pain [11], and multi-joint pain [12], which found the impact of multiple joint pain is substantial and greater than simply just an additive of combining the impact of single joint problems. The aim of this paper is to describe the prevalence and impact of foot pain on activities of daily living.

## Methods

As described previously [11], 18,827 participants over the age of 55 years were randomly selected from the North Yorkshire Family Health Services Authority (population of 210,00) and were sent a questionnaire through the post. Generic demographic information (age, gender), the presence of co-morbidities (self-reported co-morbidities that had been diagnosed by a health care professional) and activities of daily living were collected (Table 1). Participants were also asked to indicate the location of joint pain, stiffness or swelling last 3 months which had lasted for more than 6 weeks on a manikin. The foot region had two boxes: one for the foot and one for the ankle. However, following feedback from a patient focus group to explore the fidelity of interpretation for our secondary analysis, there was concern that people would not be able to discriminate pain in the ankle from pain in the foot. Therefore, we combined all foot and ankle data into one item termed ‘foot pain’ which represented foot and ankle pain. The questionnaire provided the basis of several publications including the prevalence and impact of multiple site joint pain [12]. This current study aims to determine specifically the prevalence and impact of foot and ankle joint pain.

**Table 1:** Stem questions for co-morbidities and activities of daily living and disability [12]

Co-morbidities		
Have you ever been told by a doctor or other health professional:		
That you have arthritis or rheumatism?	□Yes	□No
That you have high blood pressure?	□Yes	□No
That you have diabetes?	□Yes	□No
That you have had a stroke?	□Yes	□No
Functional ability		
In the last three months, have you ha any difficulties with any of the following activities because of health problems or disabilities?		
Gripping or holding things	□Yes	□No
Brushing or combing your hair	□Yes	□No
Getting up and down stairs	□Yes	□No
Getting up from a chair or the toilet	□Yes	□No
Putting on shoes, socks or stockings	□Yes	□No
Standing or walking	□Yes	□No

### Statistical analysis

The data were explored for non- responder bias and found those who responded were more likely to be female and slightly younger: prevalence data were therefore weighted by gender and age [12]. Unweighted data were used for all modelling and inferential analysis. The presence of foot pain, gender, age and the presence of one or more self-reported healthcare diagnosed co-morbidity, were included in a forward, step-wise regression analysis [12]. Variables were investigated for both main and interaction effects and the summative odd risks for joint combinations included the main and interaction effect of the joints involved and based on that described in the literature [13, 14]. Data were analyzed using SPSS Statistics Version 25.

## Results

Completed questionnaires were received from 16,222 people (response rate 86%). The prevalence of foot and ankle problems was 184.33 per 1000 (95%CI = ±24.033), with a greater number of women (χ^2^ = 980.994, *df* = 1, p ≤ 0.001) and older people (χ^2^ = 307.218, *df* = 2, p ≤ 0.001) reporting foot problems (Table 2). Importantly, foot and ankle pain in isolation (ie not associated with any other sites of joint pain) was uncommon, with only one in 11 of those with foot pain reporting it in the foot alone, a prevalence of only 4.87 per 1000 (95% CI = ±4.314). The most common combination of foot pain with other joints were feet and knees; feet, knees and hands; and feet knees and hips; feet and hands. Summary logistic regression tables for each of the functional indicators are included in Table 3.

**Table 2:** Prevalence estimates of joint pain, swelling and/or stiffness over the last 3 months, lasting for more than 6 weeks, age and comorbidities, per 1000 [12]

Site	Male	Female	Total
Knee	176.64 *162.62–190.66*	253.92 *238.20–269.64*	220.33 *205.26–235.40*
Foot and ankle	136.28 *123.47–149.09*	221.28 *206.19–236.37*	184.33 *170.11–198.55*
Back	134.33 *121.59–147.07*	183.48 *169.28–197.68*	162.12 *148.51–175.73*
Hip	94.43 *83.22–105.64*	151.53 *138.23–164.83*	126.71 *114.23–139.19*
Age: 55 to 64 yrs	334.67 *317.78–351.55*	389.73 *372.35–407.11*	362.73 *345.57–379.89*
65 to 75 yrs	337.26 *320.35–354.17*	401.64 *384.18–419.10*	372.49 *355.24–389.75*
75 year plus	311.56 *294.95–3 28.17*	460.80 *443.08–478.52*	409.71 *392.20–427.23*
Co morbidities	352.14 *335.08–369.20*	444.56 *426.89–462.23*	404.39 *386.91–421.87*

Estimates have been adjusted for age and gender with the upper and lower 95%confidence intervals presented in italics

**Table 3:** Summary Logistic Regression Table for the impact of site of joint pain on activities of daily living

	Variable	Co-ef (β)	Stan. Error	Wald	P	OR	95% CI
Standing and Walking							
Main effects	Constant	−2.232	0.162	–	–	–	–
	Foot and ankle	0.839	0.059	201.74	0.000	2.314	2.061 to 2.598
	Hip	0.868	0.066	172.030	0.000	2.382	2.093 to 2.712
	Co morbidities	1.751	0.134	171.872	0.000	5.761	4.434 to 7.486
	Knee	1.006	0.093	117.247	0.000	2.734	2.279 to 3.280
	Back	0.403	0.060	45.336	0.000	1.496	1.331 to 1.683
	*Foot and ankle*	*0.8745*	*0.1118*	*61.2101*	*0.000*	*2.398*	*1.926 to 2.985*
	Gender	0.945	0.191	24.501	0.000	2.573	1.770 to 3.742
	Age: 55 to 64 yrs	−0.522	0.121	18.626	0.000	0.593	0.468 to 0.752
Int	Gender (male) by age: 55 to 64 yrs	−0.628	0.151	17.389	0.000	0.534	0.397 to 0.717
	Co morbidity by Gender	−0.435	0.162	7.210	0.007	0.648	0.472 to 0.889
Going up and down stairs							
Main effects	Constant	−2.199	0.142	–	–	–	–
	Co morbidities	1.509	0.101	225.088	0.000	4.524	3.714 to 5.510
	Age: 55 to 64 years	−1.122	0.143	61.364	0.000	0.326	0.246 to 0.431
	Knee	0.710	0.098	52.093	0.000	2.033	1.677 to 2.465
	*Foot and ankle*	*0.537*	*0.075*	*51.755*	*0.000*	*1.711*	*1.478 to 1.980*
	Hip	1.021	0.162	39.974	0.000	2.777	2.023 to 3.811
	Back	0.330	0.108	9.358	0.000	1.390	1.126 to 1.717
Int	Knee by Age: 55 to 64 yrs	0.604	0.143	17.793	0.000	1.829	1.382 to 2.422
	Gender (Male) by Age: 55 to 64	−0.474	0.149	10.100	0.001	0.623	0.465 to 0.834
	Gender (Male) by back	−0.417	0.121	11.841	0.001	0.659	0.520 to 0.836
Getting up from a chair/toilet							
Main	Constant	−1.974	0.146	–	–	–	–
	Co morbidities	1.100	0.101	117.519	0.000	3.004	2.462 to 3.664
	Hip	1.623	0.196	68.783	0.000	5.066	3.453 to 7.434
	Age: 55 to 64 yrs	−1.242	0.157	62.324	0.000	0.289	0.212 to 0.393
	*Foot and ankle*	*0.363*	*0.094*	*14.999*	*0.000*	*1.438*	*1.197 to 1.729*
	Gender (Male)	0.380	0.110	11.850	0.001	1.462	1.178 to 1.814
	Knee	0.382	0.118	10.585	0.001	1.466	1.164 to 1.845
	Back	0.362	0.119	9.271	0.002	1.436	1.138 to 1.813
Int	Knee by Age: 55 to 64 yrs	0.634	0.145	19.179	0.000	1.885	1.419 to 2.503
	Back by Age: 55 to 64 yrs	0.494	0.142	12.022	0.001	1.638	1.239 to 2.165
	Gender by Age: 55 to 64 yrs	−0.525	0.150	12.237	0.000	0.591	0.441 to 0.794

Included are the summary of the main effects and interactions for each of the major sites of OA, with foot and ankle joint pain in italics. Abbreviations: co-eff (β) = the mathematical weighting of each variable in the model; Stan Error = the estimated error of the mathematical weighting; OR = odds ratio; 95% CI = the 95% confidence interval for the estimated odds ratio; int = interaction effects

As we have previously reported [12], the impact of multiple sites of joint pain is substantial: for example, those with knee and foot pain (the most common of the multiple site pain presentation) increased the difficulty of walking and standing 14-fold. As the previous publication reported the data for multiple site prevalence and impact, this paper reports the impact of foot and ankle pain, regardless of which other sites were involved and compare that to the other major sites of joint involvement.

Of note, the presence of self-reported, health care diagnosed co-morbidity had the greatest impact on activities of daily living, which we have discussed in detail in our previous publication [12]. While the importance of co-morbidity is undoubtedly substantial, care should be taken with interpreting the magnitude of the impact of co-morbidities across activities of daily living, given the potential confounding influence of co-morbidities across different activities.

For standing and walking, in addition to co-morbidities, the greatest predictor of who would report difficulty was the presence of knee pain, who were almost three times more likely to report difficulty (R^2^ = 0.324, OR = 2.734, p ≤ 0.001, 95%CI = 1.713–2.716). Of note, however, foot and ankle pain increased the risk of reported difficulty by almost two and a half times (OR = 2.314, *p* ≤ 0.001, 95%CI = 1.349–1.993), which was an equivalent risk to hip problems (OR = 2.382, *p* ≤ 0.001, 95%CI = 2.353–3.513), and higher than those who reported back problems (OR = 1.496, *p* ≤ 0.001, 95%CI = 1.5910–2.3593).

People with knee and hip problems were two and over two and a half times respectively more likely to report difficulty in climbing up or down stairs (OR = 2.033, *p* ≤ 0.001, 95%CI = 2.8012–4.2896; OR = 2.777, p ≤ 0.001, 95%CI = 2.0263–3.3062). While these were the greatest predictors of problems, those with foot and ankle problems were 71% more likely to experience difficulty (OR = 1.711, p ≤ 0.001, 95%CI = 1.9260–2.9851), which is once again, a greater risk than those who had back problems (OR = 1.390, p ≤ 0.001, 95%CI = 1.1640–1.7036).

In the task of rising from a seated position (the chair or the toilet), the presence of hip problems was the greatest predictor of difficulty (OR = 5.066, p ≤ 0.001, 95%CI = 2.2313–4.3964). Foot and ankle problems increased the difficulty in rising from a seated position to a high risk by 44% (OR = 1.438, 95%CI = 1.197–1.729, p ≤ 0.001), which was slightly more than to knee and back problems.

## Discussion

The purpose of this paper was to interrogate an existing, unique and large data set to explore the prevalence and impact of foot and ankle related pain in a community based cohort of people over the age of 55. The prevalence of foot and ankle problems was 184.33 per thousand, with a greater number of women and older people reporting foot problems which is consistent with other studies [2, 3, 5, 15–17]. Of note, foot and ankle joint pain was the third most common site of self-reported joint pain, behind only knee (220.33 per 1000) and wrist/hand pain (190.09 per 1000) as reported in our previous publication [12], and the second most common single joint presentation behind knee. Importantly, as reported in our previous paper, isolated foot related pain was relatively uncommon, with only 1 in 38 of those with foot pain having foot pain alone [12]. As described previously, the most common combination of foot pain with other joints were feet and knees; feet, knees and hands; and feet knees and hips; feet and hands; and the combination of joint problems had a substantial impact on the activities of daily living, indicating that there was an interaction effect of multiple joint pain above and beyond a simply what was seen with single site involvement [12].

As we described in our previous publication, there was a correlation between increasing number of sites of pain and impairment of daily activities. However, this was not simply an additive issue (ie the more sites of pain, the greater the impact) but an exponential increase in difficulty with different combinations of sites of joint pain. Unsurprisingly, was the joint combination that had the most substantial impact was a combination of knee, back, foot/ankle, and hip pain [12]. Other joint combinations that included the foot and ankle which increased the risk of impact on daily activities were the hip and foot, knee and foot/ankle; and knee, hip and foot/ankle. The current results and those of Finney et al. [3], indicate that specific health care attention should be paid to foot and ankle complaints in addition to other more commonly reported joint problems.

In this study, co-morbidities were found to be the single most significant predictor of who would report difficulty in walking, climbing stairs and standing. Previously, in a large UK survey, half of all severe disability in the community came from stroke and arthritis [18]. More recently, a study of those who had survived a stroke found that almost half (47%) had musculoskeletal problems [19]. In the current study, only the hip was greater than co-morbidities as the single most influential variable on rising from a seated position. While these were the greatest predictors of problems, those with foot and ankle problems were 44% more likely to experience difficulty which is a greater risk than those who had back problems. This is significant as low back pain is well established as causing more global disability than other any other condition [20].

While not the primary purpose of the initial survey, there are several key elements which support this additional analysis. First, the response rate of 86%, higher in this study than other comparable studies [2, 3, 5, 15–17] and the large cohort of people aged over 55, offers a unique and robust platform to explore the impact of foot pathology. Second, the impact of foot joint pain in either isolation, or in combination with other common joint patterns, on activities of daily living has not been previously reported. Finally, this analysis provides the contextual information of the importance of the foot in the context of other, more commonly reported joints.

The limitations of this study are acknowledged. This is a secondary analysis of a large community cohort and was not the primary purpose of the study design and also the data were collected over 20 years ago. Thus, while it is possible that the age-specific prevalence has not changed, the ageing of the population will mean that raw numbers will have increased. The issue of co-morbidities is important: as a questionnaire, self-reported health professional diagnosed co-morbidities have been include, which has limitations. Our data does, however the prevalence figures in this study are comparative to similar reports of multi-site joint pain [3]. Furthermore, we have only included a count of the number of co-morbidities: there would be great merit in looking at individual or groups of co-morbidities and their impact. Finally, we also recognize that other variables that were not considered in the logistic regression modelling may also be likely to impact on functional ability.

## Conclusion

The functional ability of people over the age of 55 is multifaceted, with the presence of existing medical problems and age influencing difficulty in undertaking daily activities. Foot and ankle joint pain are common, and contribute to functional impairment and commonly occur with other sites of joint pain. As treatments for foot pain have been demonstrated to be effective, foot and ankle problems should be considered in the overall assessment and management of impairment through appropriate multi-disciplinary approach.

## Data Availability

The datasets used and/or analysed during the current study are available from the corresponding author on reasonable request.
